# Effect of Rehabilitation of Completely Edentulous Patients With Complete Dentures on Temporomandibular Disorders: A Systematic Review

**DOI:** 10.7759/cureus.28012

**Published:** 2022-08-14

**Authors:** Sharayu Nimonkar, Surekha Godbole, Vikram Belkhode, Pranali Nimonkar, Sweta Pisulkar

**Affiliations:** 1 Department of Prosthodontics, Sharad Pawar Dental College and Hospital, Datta Meghe Institute of Medical Sciences, Deemed to be University, Wardha, IND; 2 Department of Prosthodontics and Crown & Bridge, Sharad Pawar Dental College and Hospital, Datta Meghe Institute of Medical Sciences, Deemed to be University, Wardha, IND; 3 Prosthodontics, Private Practice, Wardha, IND; 4 Lecturer, Trauma Care Center, Government Medical College and Hospital, Nagpur, IND

**Keywords:** vertical dimension, rehabilitation, edentulous, temporomandibular disorders, complete denture

## Abstract

The present study evaluates the effect of rehabilitating completely edentulous patients with complete denture prosthesis on temporomandibular disorders (TMD). The Preferred Reporting Items for Systematic Reviews and Meta-Analyses (PRISMA) standards were used to perform this systematic review, primarily to describe the technique. Manuscripts published between 1965 and December 31, 2021, were searched in the two most common electronic databases, PubMed and Cochrane Library. To find relevant scientific papers on the influence of complete dentures on temporomandibular disorders, an electronic pursuit of peer-reviewed articles confined to the English language and a dental survey were undertaken. Two observers reviewed the abstracts separately and chose five full-text papers that met the inclusion requirements. Due to the heterogeneity of the data provided, a meta-analysis could not be performed. The result of complete dentures on temporomandibular problems was studied in detail in five peer-reviewed papers. The result of the present study concluded that the complete denture could act as a conducive treatment option to the revocation of TMD for elderly edentulous patients.

## Introduction and background

Temporomandibular disorder (TMD) is a comprehensive term that refers to problems with the temporomandibular joint (TMJ), masticatory muscles, and occlusion. The most common symptoms are joint noises, TMJ pain, masticatory muscle tightness, and restricted lower jaw movement [1].

The specific cause of TMD is yet unknown. TMD, on the other hand, has been linked to behavioral, psychological, and physical variables [2]. In several studies, TMD has been related to occlusal disparity and the absences of posterior teeth in totally or partially edentulous patients [3]. At the same time, there is evidence of a significant frequency of TMD in totally edentulous patients [4]. This added to the debates and the need for more research in the field of TMD.

De La Torre Canales et al. observed severe somatization and depression in patients with TMD in a systematic review [5]. TMD symptoms influence oral health-related quality of life and psychological suffering. TMD has been linked to a lower quality of life, according to Tay et al. and Bitiniene et al. [6,7].

Early diagnosis of TMD signs and symptoms in asymptomatic patients can help prevent the problem from developing. This systematic study examined the impact of restoring lost vertical dimension and centric relationship in edentulous individuals with complete dentures on the severity of TMD signs and symptoms.

## Review

Materials and methods

This systematic review was executed according to the Preferred Reporting Items for Systematic Reviews and Meta-Analyses (PRISMA) guidelines [8]. The Population, Intervention, Comparison, Outcome (PICO) structure was used to create a focal question, and a systematic search strategy for the study was defined as a result (Table 1).

**Table 1:** Systematic search strategy TMD: temporomandibular disorders

Systematic search strategy	Protocol followed
Focus question	Population -Patients with temporomandibular disorders Intervention Complete denture prosthesis or implant-supported overdenture Comparison Advantage of complete denture wearing in reducing TMD over non-wearers Outcome To assess the advantage of using complete denture or implant-supported overdenture on the temporomandibular joint.
Search combination	“completely edentulous" AND “Temporomandibular disorders” OR "complete Denture" AND “Temporomandibular dysfunction”
Electronic database searched	PubMed/Medline, Cochrane library
Inclusion criteria	Language of publication English Article types Clinical Trials Articles related only to the assessment of changes in TMDs after treatment with complete denture prosthesis
Exclusion criteria	Language of publication other than English Articles related to treatment options other than a complete denture for completely edentulous patients.

Review question

The search was framed by the PICO question, where the population was the patients with completely edentulous arches having signs and symptoms of TMD. The intervention was rehabilitation with a complete denture or implant-supported overdenture using various occlusal schemes and variations in the clinical steps of complete denture fabrication. The comparison was between denture wearers and non-wearer over the intensity of signs and symptoms of TMDs. The primary outcome was to determine whether the signs and symptoms of TMDs improve after using a complete denture prosthesis, and the secondary outcome was to analyze the severity of TMDs in complete edentulous subjects, to determine the changes in the grade of TMDs after the use of complete denture and to determine the specific changes in clinical steps of denture fabrication that can contribute to reducing TMD.

Literature search

For manuscripts published between 1965 and December 31, 2021, two electronic databases, PubMed and Cochrane Library, were looked at. Two independent reviewers were assigned to filter the titles and abstracts. The complete text of articles that met the requirements for inclusion was collected. The ultimate search was made manually from the selected articles for cross-references and citations to incorporate all relevant papers and improve the electronic search. Following an electronic and manual search, PubMed returned 120 publications, but the Cochrane Library revealed no systematic reviews on the subject. There were 22 articles found in advance search for the Medical Subject Headings (MeSH) terms "completely edentulous" AND "Temporomandibular disorders" OR "complete Denture" AND "Temporomandibular dysfunction". Only 11 papers were found to be pertinent to this issue and met the inclusion criteria for the systematic review after a thorough examination of their titles and abstracts.

Results

Results of Data Extraction

**Figure 1:**
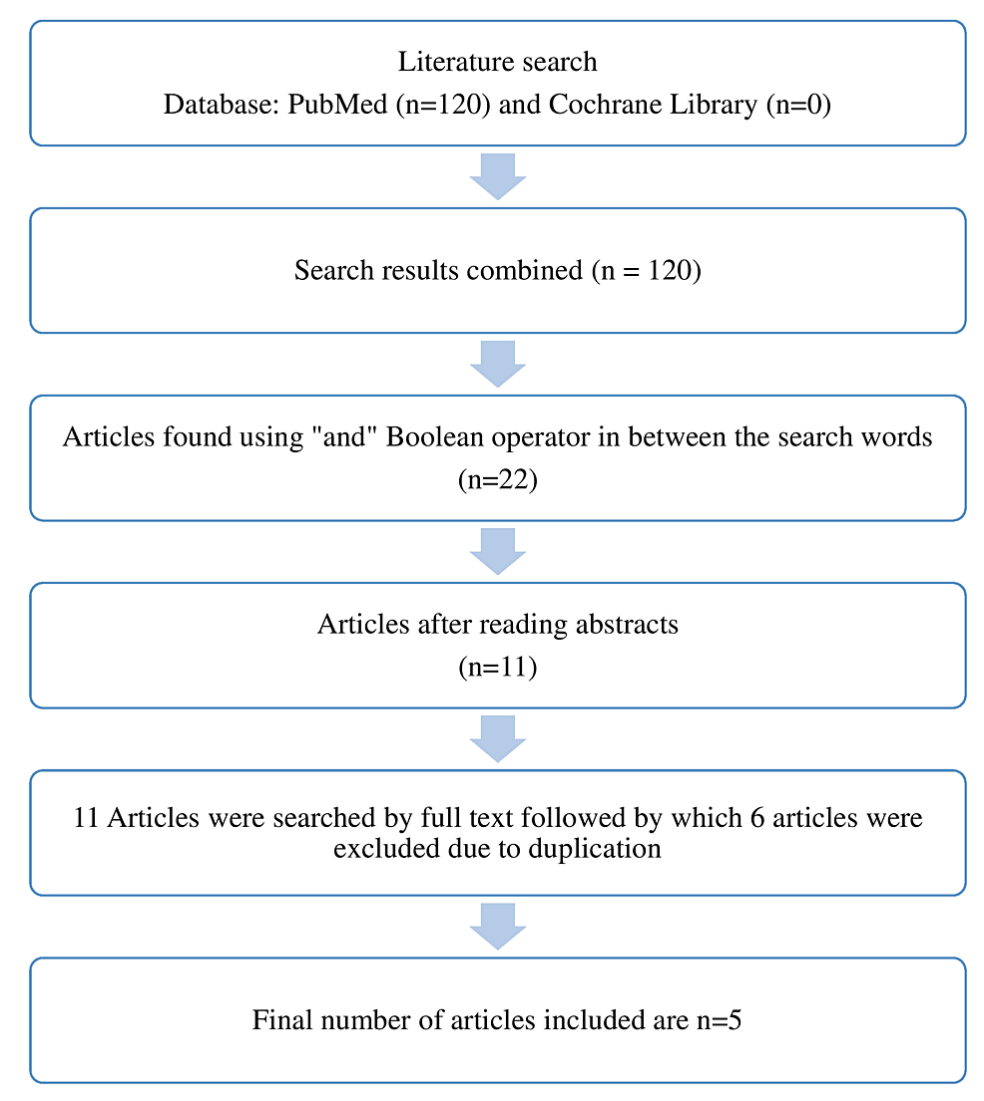
Flowchart of the studies

The complete text of these 11 publications was retrieved, and the two independent reviewers did an in-depth review for each of these 11 articles, out of which six were deleted due to duplication. As a result, the sample size for this systematic review was determined to be five articles (Figure 1).

Results of Included Studies 

The studies involving complete denture as a treatment therapy for treating TMDs that were covered in the present systematic review are the studies by Engel et al. in 1995 [9], Wolfart et al. in 2005 [10], Zuccolotto et al. in 2007 [11], Yu et al. in 2013 [12] and Nitecka-Buchta et al. in 2018 [13] (Table 2).

**Table 2:** Studies included in the systematic review TMD: temporomandibular disorders, EMG: electromyography

Author	Title of article	Year	Conclusions of the studies
Engel E et al. [9]	Treatment of edentulous patients with temporomandibular disorders with implant-supported overdentures	1995	Subjects treated with implant-supported overdentures were interviewed and subjected to clinical, functional analysis that showed a reduction in TMJ noises
Wolfart S et al. [10]	Effects of prosthetic treatment for shortened dental arches on oral health-related quality of life, self-reports of pain and jaw disability: results from the pilot phase of a randomized multicentre trial.	2005	Prosthetic rehabilitation with overdentures showed positive effects on Jaw Disability Checklist determined by Research Diagnostic Criteria and improved life quality by Oral Health Impact Profile
Zuccolotto MC et al. [11]	Electromyographic evaluation of masseter and anterior temporalis muscles in the rest position of edentulous patients with temporomandibular disorders, before and after using complete dentures with sliding plates.	2007	EMG analysis after prosthetic rehabilitation showed reduced stomatognathic pain, remission of muscular weariness, and neuromuscular deprogramming in TMD
Yu TH et al. [12]	Clinical research on curative effect of complete denture with two kinds of occlusion for temporomandibular joint disorders of aged edentulous patients.	2013	Lingualized occlusion has increased TMD remission in edentulous patients
Nitecka-Buchta A et al. [13]	Functional Assessment of the Stomatognathic System, after the Treatment of Edentulous Patients, with Different Methods of Establishing the Centric Relation	2018	The gothic arch tracing technique of establishing centric relation yielded a superior effect in TMD patients over the other conventional methods

Discussion

The signs and symptoms of TMD are directly linked to tooth loss. TMD is quite common and severe among the entirely edentulous population. It has been noted that changes in different angles caused by absences of the tooth impact TMJ mechanics, resulting in TMD. Due to the loss of vertical dimensions, the mandible shifts from its actual centric relation to a continual centric position when a complete denture prosthesis is not worn for a long time [14]. Similarly, psychological issues related to tooth loss, aging, and emotional stress all can develop TMD in the edentulous population [15]. The prevalence of TMD in edentulous patients was investigated by Shetty [16]. He found that 59% of the study participants had more than two indications of TMD. Meyerowitz et al. examined the masticatory muscles and observed that 32% of their study subjects reported pain [17]. In 2019, AlZarea observed a significant frequency of TMD (60.5%) in asymptomatic, fully edentulous patients [18]. Shi et al. also reported symptoms of TMD in 43.2% of the study individuals (totally edentulous subjects) [19].

TMD is identified clinically by assessing the following signs and symptoms: facial pain, headache, joint pain, pain that gets worse when you open your mouth, muscle tenderness, pain in the lower jaw angle and cervical muscles, reduced opening of the mouth, jaw deflection while mouth opening, crepitus, and clicking sound in the TMJ. The indicators of TMD can emerge in a variety of ways and to varying degrees. TMJ noise was shown to be a common symptom by Shi et al. [19], although joint sounds were determined to be the least common by AlZarea [18] in his study. Shetty [16] reported similar results, with the joint sounds being the most usual symptom in 47% of the cases.

Personal care, physical cure, splints, physiotherapy, behavioral remedy, relaxation approaches, muscle-relaxing products, and pharmacological therapies are among the most common treatment options for TMD [20]. Along with these well-made complete dentures, restoring lost vertical dimension and correct centric relation is vital to support muscles, improve coordination, and prevent muscular spasms.

Various case reports and clinical trials are documented regarding the effect of restoring completely edentulous patients with complete dentures on temporomandibular disorders [21]. However, no systematic review has been conducted on this particular subject yet. This systematic review concentrated on the benefits of removable complete denture prosthesis among completely edentulous subjects in improving the condition of TMDs. This systematic review included articles published between 1965 and December 2021 that met the study's inclusion criteria. 

In 1995, Engel et al. used implant-supported overdentures to treat edentulous patients with temporomandibular disorders [9]. They discovered that treating edentulous individuals with TMJ disorders is challenging since their conventional complete dentures are unstable, which can be improved with an implant-supported overdenture. Ten edentulous patients with TMJ problems were treated with implant-supported overdentures in a prospective clinical investigation. The study participants were subjected to an interview and a clinical functional analysis before and after using three years of an implant-supported overdentures prosthesis. Patients with articular disc displacement or joint bone degradation experienced minor discomfort, increased mandibular mobility, and less temporomandibular joint noises.

The effects of prosthetic rehabilitation with small dental arches on self-assessment of pain and jaw impairment were researched by Wolfart et al. in 2005 [10]. They tested 34 people who were missing all of their molars and had not less than both canines and at least one premolar in every four quadrants. Removable partial dentures in the form of a tooth-supported overdenture or retained premolar occlusion were given to participants randomly. Participants filled the Oral Health Impact Profile and the Research Diagnostic Criteria for TMD before and after therapy at six weeks, six months, and 12 months. The study's findings demonstrated an improvement in life quality and only a considerable reduction in pain. The Jaw Disability Checklist had a slightly larger number of effects.

In 2007 Zuccolotto et al. performed an electromyography (EMG) evaluation of masticatory muscles in the rest position among the completely edentulous subjects with TMD, before and after using complete dentures having sliding plates [11]. They used computerized electromyography EMG to record EMG before and after the insertion of the dentures, as well as after employing the sliding ramp/plates at the fourth, ninth, and 12th months. All the patients had reduced pain in the stomatognathic system structures and remission of muscular weariness. They found that the sliding plates enabled neuromuscular deprogramming, which contributed to masticatory system muscle balance. They should be utilized before manufacturing final complete dentures in subjects suffering from TMD.

Yu et al. published a clinical study in 2013 that looked at the outcome of a complete denture with two types of occlusion scheme on the temporomandibular joint abnormalities in elderly completely edentulous subjects [12]. On TMD with substantial residual alveolar ridge degradation, he evaluated the therapeutic impact of complete dentures with lingualized occlusion versus anatomic occlusion. The Fricton Index was used to assess TMD's recovery effect. The craniomandibular index revealed a lower value (0.064 0.022) for lingualized occlusion dentures after three months but considerably higher values for anatomic occlusion (0.043 0.018) (P 0.01). The findings concluded that full dentures with lingualized occlusion might increase TMD remission in elderly edentulous patients with severe residual alveolar ridge erosion.

In 2018, Nitecka-Buchta et al. evaluated the stomatognathic system following edentulous patients' treatment with various techniques of establishing the centric relation [13]. The study compared patients' subjective experiences with complete dentures. They used two approaches to determine a centric relation: the old method, which used wax occlusal rims, and the Gerber method, which relied on gothic arch tracings. The gothic arch tracing method yielded a superior outcome.

There are currently inadequate randomized clinical trials to demonstrate the efficacy of existing medications for treating TMD in totally edentulous people. More research is needed to examine the capability of precisely fabricated complete dentures in alleviating the signs and symptoms of TMD in a completely edentulous population. This will aid in developing a plan for early intervention in totally edentulous patients with complete dentures to impede the worsening of joint complications and asserts orofacial musculature harmony.

## Conclusions

Presently, there is no systematic review published on the accessible literature debating the effect of complete dentures on temporomandibular disorders. This systematic review was undertaken to justify whether edentulism rehabilitated with a complete denture can minimize the severity of the TMDs.The studies in the present systematic review concluded that restoring loss of vertical dimension and correct centric relation in completely edentulous patients improves the TMD. The slight changes in methods from conventional and sometimes the more proper traditional approaches will undoubtedly enhance the condition of elderly patients. The complete denture not only serves the purpose of replacement but also has a curative effect on the stomatognathic system. Timely identification and managing this condition may improve the quality of life and provide primary care for such patients.
